# Biodegradable Temporizing Matrix (BTM) resilience to wound infection: A consecutive case series

**DOI:** 10.1016/j.jpra.2025.07.007

**Published:** 2025-08-19

**Authors:** Sasha Wilson, Edward Muscat, Olivia Smith, Alex Noakes, Christopher Wearn

**Affiliations:** Department of Plastic Surgery, Southmead Hospital, Southmead Road, Bristol, BS10 5NB, United Kingdom

**Keywords:** NovoSorb® Biodegradable Temporizing Matrix, BTM, Infection, Colonization, Complex wounds, Wound healing

## Abstract

PolyNovo® Biodegradable Temporizing Matrix (BTM) has gained favor for reconstructing non-graftable wound beds amid suggestions it is inherently resistant to infection. Evidence is limited to multiple case series and currently the rates of BTM colonization, infection and loss remain unestablished. We report our case series which includes a diverse case-mix of complex wounds successfully reconstructed with BTM, with a novel focus on the rate of infection and related microbiology profile. Our case series included all patients treated with BTM at Southmead Hospital between October 2021 and November 2023. In our cohort, 22 patients had their wounds reconstructed with BTM. Microbiological growth was observed in 76 % of available pre-BTM swabs and subsequently only 14 % (3/22) of all patients experienced clinical infection of BTM. In the case of infection, 66 % (2/3) of patients had successful salvage of BTM. Only one patient (5 %) in the cohort experienced partial BTM loss, with the remainder achieving total wound healing at 3 months. Our study makes a case that microbiological wound swabs prior to BTM application is of limited clinical value. In our experience, salvage techniques can be applied empirically in the case of clinical infection and the vast majority of patients will have successful integration of the BTM.

## Introduction

NovoSorb® Biodegradable Temporizing Matrix (BTM) is a novel, fully synthetic bi-layer skin substitute made from a 2 mm thick polyurethane foam scaffold and a non-biodegradable outer sealing membrane.^1^ It was initially developed for use in burn surgery and, more recently, it has gain traction in the two-stage reconstruction of complex wounds following trauma, oncological resection or infection.

Preliminary animal studies of BTM highlighted that it successfully heals wounds with less contraction than a competing bi-layer skin substitute, Integra®.^2^^,^^3^ Studies suggest that BTM is inherently resistant to infection when compared to Integra®.^3^ In-vivo human studies observed the evolution of applications of BTM to reconstruct clean surgical wounds followed by its successful ability to reconstruct a non-graftable calvarial wound from major burn injury.^4^^,^^5^ These early studies highlight that BTM can covert non-graftable wounds into graftable wound beds through cultivation of a vascularized neo-dermis and, crucially, that it can achieve wound healing even in the presence of infection.^5^^,^^6^ BTM’s observed resistance to infection is theorized to be due to its fully synthetic composition which does not provide a substrate for microorganisms grow, unlike biological agents.^1^ Additionally, the sealing membrane mimics the function of the epidermis and provides a physical barrier to microorganism entry into the wound.^1^

In modern practice, BTM is increasingly used to reconstruct non-graftable wounds at increased risk of colonization or infection, such as those created by trauma.^6^,^7^ Wagstaff et al.^.^^6^ highlighted that, in the presence of infection, BTM will continue to integrate into the wound bed as long as any collections are drained through perforations in the sealant membrane. The robustness of BTM to infection was studied further in 2023, as Guerriero et al.^8^ reported that it can successfully integrate into complex diabetic foot wounds, even in the presence of pre-existing clinical infection.

### Rationale

The clinical applications of BTM are rapidly expanding and this is reflected in a surplus of published case series surrounding its novel applications to reconstruct traumatic wounds.^9^ In an attempt to consolidate the evidence, two systematic reviews have recently been published, summarizing the key findings from a wide range of heterogeneous studies investigating BTM’s utility in managing both burns and traumatic wounds.^10^, ^11^, ^12^ Fruergaard et al.^12^ summarizes data from 880 patients, however 40 % of these patients lack data on the rate of infection and 25 % lack data on the rate of BTM loss. This reflects that the current body of evidence is lacking data regarding complications following BTM reconstruction. Such data would be useful to inform clinicians starting to use the product on how it can be best employed and enable more detailed information to be given to patients when consenting for this type of reconstruction.

### Objectives

Our primary aim is to add to the current body of evidence surrounding BTM’s use in managing complex traumatic wounds, focusing specifically on the incidence of infection and BTM loss, by reporting the findings of an early consecutive case series from a tertiary plastic surgery service in Bristol, United Kingdom.

## Methods

Using a prospectively maintained database, we conducted a retrospective consecutive case review of all BTM reconstruction at a single tertiary center for plastic surgery at Southmead Hospital, Bristol, UK. The study period was 25 months, from October 2021 to November 2023. All patients were discussed at a Trauma Meeting with at least one consultant present; alternative reconstruction options were considered before the decision for reconstruction with BTM was agreed. All consecutive patients were included, with no exclusion criteria applied.

The typical management of cases in the cohort was relatively standardized as follows. Reconstruction when macroscopically clean wound and no further debridement needed. Application of silver based dressings, typically Acticoat or Acticoat Flex, in combination with Topical Negative Pressure (TNP) therapy. In the absence of TNP, compressive bandaging was used where possible. BTM was secured with staples or non-absorbable sutures in all cases. Inspection for BTM integration, vascularization and complication was performed at every dressing change. Second stage was performed once the vast majority of the BTM was fully integrated as demonstrated by 1) No visible matrix 2) Uniform dark pink coloration 3) Uniform capillary refill 4) Fullness of the matrix on palpation. BTM was cleaned at each dressing change with aqueous betadine or chlorhexidine solution. When ready for second stage, BTM was delaminated and the integrated matrix outer layer cleaned with antiseptic and freshened with diathermy scratch pad. Split thickness skin graft (SSG) of 8–10/1000” thickness, either fenestrated or meshed, were applied and secured with staples or sutures. Grafts were dressed with Jelonet, betadine soaked dressing gauze, dry dressing gauze and bandaging or a non-adherent silicone based dressing underneath TNP.

Data collected for the primary outcome measure was complete wound healing. This was defined as >95 % epithelization from the original wound site. The main secondary outcome was incidence of BTM infection before SSG application. We defined localized BTM infection in the presence of erythema surrounding the edge of the wound and/or producing suppurative or purulent discharge underneath the BTM seal. Systemic features of infection were also recorded such as pyrexia or haemodynamic instability in the context of a locally infected wound. To record this accurately, microbiological wound swabs taken before application of BTM (pre-BTM), before delamination and SSG (pre-SSG) and from clinically infected BTM wounds were included. Other measured outcomes included: reason for admission, indication for BTM, length of hospital stay, duration of TNP therapy, SSG take and any complications**.** Data were collected, and patient care was reviewed over a minimum of 3 months after second-stage reconstruction with SSG. Demographic data such as patient age, smoking status, comorbidities, number of wounds and wound total body surface area (TBSA) were also collected.

Clinical data was extracted retrospectively from the patient’s electronic patient record (EPR) and inputted onto a Microsoft Excel® spreadsheet (Microsoft Corporation, USA) prior to statistical analysis. No formal ethical approval was required as this was a retrospective observational study. All clinical photography included required patients’ written consent for use in publication.

## Results

### Overall demographics

In total, 22 patients were included in this study with 30 wounds treated. 77 % (n = 17) of patients had a single wound whereas 23 % (n = 5) had multiple wounds. Median age was 48 and median ASA grade was 2. In terms of risk factors for delayed wound healing, 27 % (n = 6) were smokers, 45 % (n = 10) were non-smokers and 5 % (n = 1) were ex-smokers (Table 1).

**Table 1:** Summary data for patient cohort.

No.	Age	ASA	Smoking (Y/N/Ex)	Wound aetiology	Reason for BTM	Wounds (n)	Total Wound(s) size (TBSA %)	Location	Average time from BTM to SSG (days)	Pre-BTM swab	Pre-SSG swab	BTM loss	BTM Clinical infection	Complication
1	57	3	Ex	Burn + crush	Tendon exposed	4	0.25	Hand/wrist	30	Positive	Positive	Y (2/4 wounds)	Y (IV abx)	Y (tendon exposure)
2	57	3	N	Secondary to infection (Necrotising fasciitis)	Major skin loss	1	13	Leg & thigh	28	Positive	Positive	N	N	N
3	70	2	N	Burn	Tendon exposed	2	0.5	1) Hand 2) Wrist	35	Positive	Positive	N	N	N
4	32	2	Y	Open fracture (trauma)	Bone exposed	1	0.75	Foot/ankle	33	N/A	N/A	N	N	N
5	33	1	Y	Open fracture (animal bite)	Tendon exposed	1	0.75	Foot/ankle	34	Positive	Positive	N	N	N
6	66	3	Y	Burn	Bone exposed	1	0.5	Hand/wrist	66	Positive	Positive	N	N	Y (bone spicule excised)
7	36	1	N	Secondary to infection (necrotising fasciitis)	Tendon exposed + major skin loss	1	4	Arm	41	Negative	Positive	N	N	N
8	32	1	N	Secondary to infection	Tendon exposed	1	0.25	Hand/wrist	58	Negative	N/A	N	N	Y (Early delamination)
9	29	3	Not documented	Degloving (trauma)	Major skin loss	2	10	1) Arm 2) Thigh	Not Documented	Positive (thigh)	N/A	N	N	Y (Partial SSG loss)
10	73	3	N	Secondary to infection (necrotising fasciitis)	Major skin loss	1	8	Leg	59	Negative	Positive	N	N	N
11	28	2	N	Secondary to infection (abscess)	Tendon exposed	1	0.25	Foot/ankle	35	Positive	Positive	N	Y (PO abx)	N
12	58	3	Not documented	Secondary to infection (septic emboli causing ischaemia)	Major skin loss	2	9	1) Right leg 2) Left leg	28	Positive	Positive	N	N	N
13	48	2	N	Degloving + crush	Tendon exposed + Major skin loss	1	0.75	Hand/wrist	29	Positive	Positive	N	Y (PO abx)	Y (Early delamination)
14	48	4	N	Secondary to infection (skin loss and intra-abdominal sepsis requiring laparotomy)	Major skin loss	3	8	1) Abdomen 2) Right hip 3) Left hip	30	Positive	Negative	N	N	Y (Partial SSG loss)
15	59	2	Not documented	Secondary to infection (necrotising fasciitis)	Contracture	1	0.5	Arm	35	N/A	N/A	N	N	N
16	76	2	N	Degloving + crush	Major skin loss	1	1	Foot/ankle	49	N/A	N/A	N	N	N
17	22	1	Not documented	Secondary to infection (animal bite)	Tendon exposed	1	1	Leg	44	Negative	Negative	N	N	N
18	40	3	N	Burn	Contracture	1	4	Neck	27	Positive	Positive	N	N	N
19	37	1	Y (20 pack year)	Degloving + crush	Tendon exposed	1	0.5	Hand/wrist	46	Positive	N/A	N	N	Y (Partial SSG loss)
20	58	Not documented	Not documented	Degloving + crush	Major skin loss	1	3	Forearm	43	N/A	N/A	N	N	N
21	52	3	Y (quit <2/12)	Extravasation	Tendon exposed	1	0.5	Hand/wrist	31	Positive	N/A	N	N	Y (Partial SSG loss)
22	37	4	Y	Crush	Bone exposed	1	0.5	Foot/ankle	55	N/A	N/A	N	N	Y (Partial SSG loss)

### Wound location, aetiology, and complexity

**Figure 1:**
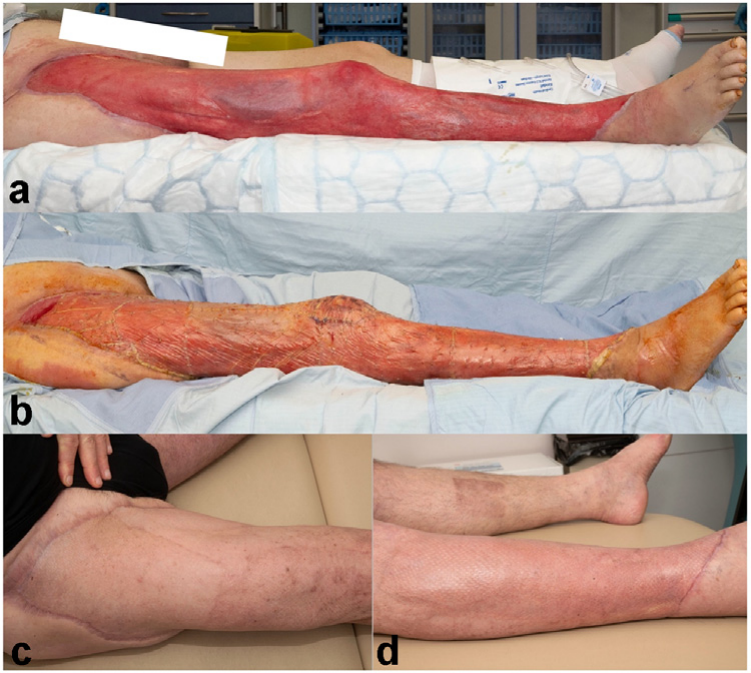
Patient 2: wound secondary to debridement of necrotizing fasciitis reconstructed with BTM. (a) Post debridement, (b) During BTM integration (2.5 weeks), (c) and (d) following 2nd stage (SSG) reconstruction (3.5 months).

**Figure 2:**
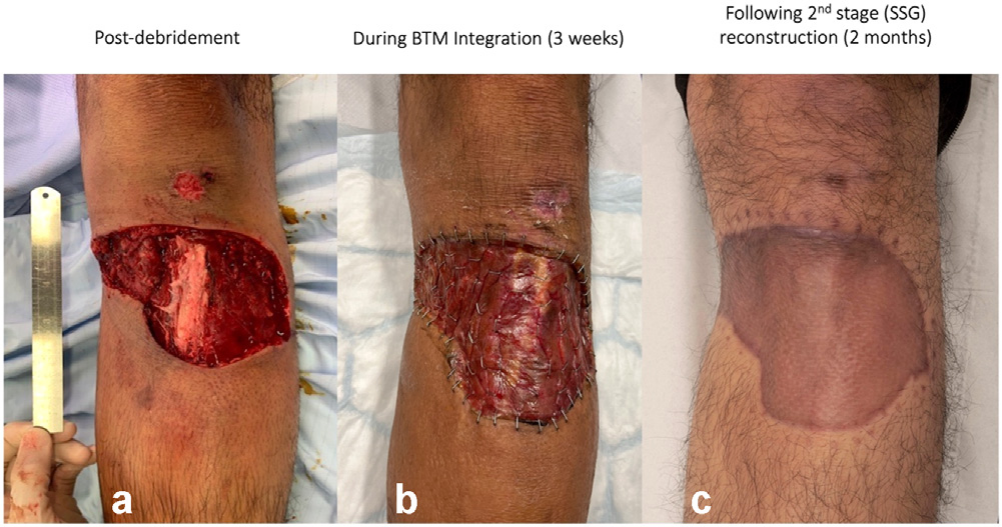
Patient 17: wound secondary to animal bite. (a) Post debridement, (b) During BTM integration (3 weeks), (c) following 2nd stage (SSG) reconstruction (2 months).

In terms of anatomical location, 45 % (n = 10) of wounds occurred on the upper limb, 41 % (n = 9) occurred on the lower limb and 14 % (n = 3) occurred either in head and neck region or multiple sites. The commonest wound aetiology was soft tissue loss secondary to infection, in 41 % (n = 9) of patients, as illustrated by Figure 1. In terms of complexity, 59 % (n = 13) of patients required reconstruction with BTM due to bone or tendon exposure in the wound bed, as illustrated by Figure 2 and Table 2.

**Table 2:** Summary of wound location, aetiology, and deep structure exposure within cohort.

	Wound secondary to infection	Traumatic crush or degloving	Burn	Open fracture	Extravasation
Number of patients	9 (41 %)	6 (27 %)	4 (18 %)	2 (9 %)	1 (5 %)
	Exposed tendon	Exposed bone	Mixed deep structure exposure	Major skin loss	Contracture release
Number of patients	8 (36 %)	3 (14 %)	2 (9 %)	7 (32 %)	2 (9 %)
	Upper limb	Lower limb	Anterior trunk	Head & neck	Multiple locations
Number of patients	10 (45 %)	9 (41 %)	1 (5 %)	1 (5 %)	1 (5 %)

### Timings of two-stage wound reconstruction

BTM application was combined with TNP therapy in 82 % (n = 18) of cases. All cases underwent second-stage reconstruction with SSG. The median time from BTM to SSG application was 35 days (27–66) (Table 3).

**Table 3:** Summary of wound size and length of treatment.

	Median	Range
Time from admission to BTM (days)	7	0.5–52
Time from BTM to SSG (days)	35	27–66
Length of stay (days)	12	0.5 –87
TBSA (%)	0.75	0.25–13
Duration of TNP (days)	8	5–85

### Primary outcome

The majority (95 %, 21/22) of patients achieved complete wound healing by 3-months with only one patient (5 %) experiencing BTM loss. Five patients (23 %) experienced some degree of SSG loss and the average SSG take was 91 % for the whole cohort.

### Secondary outcome

All wounds were macroscopically clean prior to swab and BTM application. Pre-BTM swabs were positive for 59 % (n = 13) patients and negative for 18 % (n = 4) patients; the remaining 23 % (n = 5) of patients did not have swab results available in our institution. This result highlights that 76 % of available pre-BTM swabs showed colonization with bacteria. Similarly, 85 % of the 13 available pre-SSG swabs grew bacteria, suggesting high rates of colonization.

Localized BTM infection was identified in three (14 %) of patients. Table 4 further describes the infected patients swab results pre-BTM, during infection and pre-SSG. For those who developed infection, 100 % had positive pre-BTM swabs. Microbiological samples from infected patients showed mixed growth in samples in all patients. Two patients had Staphylococcus aureus growth in at least one of the three swab cultures. All of these patients (3/3) had TNP therapy applied to BTM.

**Table 4:** Microbiology relating to cases of infected BTM.

Patient	Mechanism of injury	Pre-BTM swab	Infected BTM swab	Pre-SSG swab
1	Crush injury (hand)	1) Heavy growth (++) of Coagulase Negative Staphylococcus 2) Heavy growth (++) of Diphtheroids	1) Heavy growth (++) of Coagulase Negative Staphylococcus 2) Heavy growth (++) of Diphtheroids	1) Direct and Enrichment growth Staphylococcus simulans 2) From enrichment culture only: Staphylococcus aureus 3) Direct culture Heavy growth of Staphylococcus aureus 4) Direct culture Scanty growth of Enterobacter cloacae complex
11	Recurrent abscess (foot)	Direct culture Moderate growth Klebsiella aerogenes	1) Heavy growth (++) of Enterococcus species 2) Moderate growth (+) of Coliform x2 types 3) Moderate growth (+) of Pseudomonas species	1) Heavy growth (++) of Enterococcus species 2) Moderate growth (+) of Coliform x2 types 3) Moderate growth (+) of Pseudomonas species
13	Crush injury with degloving (hand)	1) Ochrobactrum intermedium 2) Clostridium species	Staphylococcus aureus	Heavy growth Staphylococcus aureus

**Figure 3:**
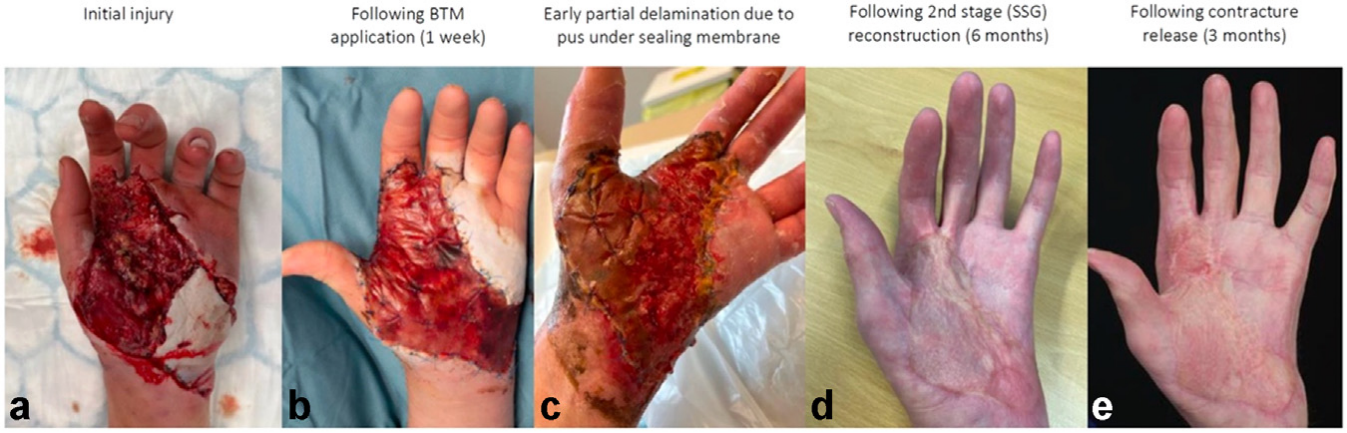
Patient 13: wound secondary to crush injury resulting in significant structural damage and palmar degloving. Reconstruction with BTM achieved complete wound healing despite BTM infection which resolved with oral antibiotics and early delamination of BTM. As illustrated, a contracture was evident at 7 months post-BTM. The contracture was released and reconstructed with FTSG and local flap at 11 months post-BTM reconstruction. (a) Initial injury, (b) Following BTM application (1 week), (c) Early partial delamination due to pus under sealing membrane, (d) following 2nd stage (SSG) reconstruction (6 months), (e) following contracture release (3 months).

Only one patient (33 %) with infected BTM experienced BTM loss, as illustrated by Figure 3. This patient experienced a combined crush and thermal injury to the right and middle fingers with associated full thickness burns and exposed tendon after debridement. An attempt was made to salvage the BTM in this case by regular “milking” of the BTM, intravenous antibiotics and iodine based dressings. This was unsuccessful, and the patient eventually required a central slip tendon repair and reconstruction with a local flap. It is worth noting that the volar surfaces of these digits had intact BTM and proceeded to a successful second stage with SSG. This patient was an ex-smoker and was the only patient from the series that required a short stay admission for intravenous antibiotics.

With regards to the other two cases of clinical infection of BTM, one had fluid expressed from under the BTM and one required early delamination. All patients were given a course of oral antibiotics according to Trust guidelines. Ultimately, they all had full integration of BTM and all proceeded to have SSG reconstruction. These formerly infected BTM cases all had pre-SSG microbiology swabs and all showed growth of bacteria as shown in Table 4. Despite the microbiological findings, there were no cases of SSG loss to any of the infected BTM cases with all cases achieving 100 % graft take.

In terms of wound complexity in patients with BTM infection, 66 % (2/3) patients had wounds secondary to infection and 33 % (1/3) associated with crush mechanism of injury. All patients (3/3) had exposed tendon in the wound base and 33 % (1/3) were upper limb and 66 % (2/3) were lower limb. The majority (2/3), were non-smokers and one patient was an ex-smoker.

### Complications

One patient was deceased several months after being discharge from hospital due to complex co-morbidities. Complications specifically related to BTM were as follows; two patients required early delamination of BTM (one due to stitch sinuses and one due to localized pus under the seal).

## Discussion

### The Existing Literature

We conducted a review of the literature surrounding the use of BTM to reconstruct exclusively non-burn wounds and results can be seen in Supplementary Sheet 1.0. In summary, nine studies included results of BTM reconstruction in 148 patients. The median number of patients was 14 (2–37). Median TBSA was <1 %, ranging from <1 to 24 %. Median time from BTM to grafting was 38.5 days. Full integration of BTM reported as 94.3 % (65.3–100 %) of all wounds treated. Total BTM loss reported as 3.7 % (0–34.7 %) of wounds. Most notably, positive pre-BTM swab results were observed in 58.8 % of wounds. BTM clinical infection rate across these studies was median 0 % of wounds, however the range was vast (0–18.9 %).

Most recently, a 2025 systematic review and meta-analysis by Grande et al.^11^ summarizes data pertaining to 202 patients, most commonly with burns injuries. Grande et al.^11^ report that 76.6% of patients did not experience infection and infected wounds were associated with delays between BTM and grafting. In addition to these studies, a single-center study of 300 patients treated with BTM was published in 2024 by Tapking et al.^13^ and was the first to provide statistically significant results that positive pre-BTM swabs and prolonged trauma-to-BTM interval are a predictors of poor BTM take. However, the findings of this study must be interpreted with care as 59.7 % of patients were being treated for burn injury which brings unique challenges relating to complications such as wound colonization and infection given the risk of immunocompromise in burns patients.

### Colonization

BTM’s fully-synthetic properties does not negate the presence of microbes, as exemplified by a high rate of positive wound swab in our case series. This is echoed by our literature review findings and the 300-patient study by Tapking et al. (2024) which reported positive wound swabs in 35.7 % of patients and full BTM integration in 82.7 %.

The difficulty in determining the utility of wound swab results in macroscopically clean wounds is further exemplified by the findings of our case series which reports positive pre-BTM wound swabs in 76 % of available swabs, with only 23 % (3/13) of these positive wound swab patients going on to develop a clinical infection.

This suggests that the mere presence of positive wound swabs does not infer high rate of infection. This does raise further questions regarding how clinicians should manage positive pre-BTM swabs in macroscopically clean wounds, as pursuit of swab negative wounds may add delays in treatment, which Tapking has also shown may adversely affect BTM take. In our unit, the current standard of practice is to carry out microbiology swabs to wound prior to BTM application. The results are used for discussion with microbiologists to inform peri-operative antibiotic cover, which normally consists of two doses.

### Clinical Infection and salvage techniques

In our case series, 14 % (n = 3) of patients developed clinically evident infection with only 5 % (n = 1) of patients progressing to partial BTM loss. The presence of BTM infection is highly variable within the available literature we reviewed, which reports a range of 0–18.9 % in available studies looking at non-burn wounds. Further to this, the median rate of BTM loss within these studies is 3.7 % however this is highly variable between studies (0–34.7 %). Unfortunately, the large case series by Tapking et al.^13^ does not report the rate of infection within their 300-patient cohort. Fruergaard et al.^12^ 2025 systematic review of BTM use in 808 patients with both burn and non-burn wounds, has a reported infection rate of 10 % and reports that infection is the most common reason cited for BTM loss.

In comparison, Integra is a non-synthetic bi-layer skin substitute, with FDA-reported infection rates of 20.5 % (26/127) and partial/total loss rates of 12.6 % (16/127).^14^ Further to this, a 2020 systematic review of Integra reconstruction by Gonzalez et al.^15^ reported an infection rate of 16.9 % (212/1254).

The results from our case series and the current body of literature highlight that BTM can become infected at clinically significant rates, however appears to become infected less frequently than Integra. Importantly, BTM appears to be robust in the face of infection, as reflected by the low reported rates of BTM loss. This notion of inherent robustness is supported by S Mason et al.^16^ in a paper directly comparing different dermal templates. Additionally, our case series reflects the ability to successfully salvage infected BTM in the majority of cases, utilizing regular evacuation of exudate, antiseptic cleaning, early delamination or windowing of BTM when needed and systemic antibiotic therapy.

### Pattern and rate of BTM Loss

As previously mentioned, the only case of BTM loss in our cohort was a complex hand injury caused by combined crush and burn mechanism. The resulting defect was two small dorsal wounds to index and middle fingers overlying the PIPJs and two wounds to the palmar aspect in a similar location. The underlying wound bed contained extensor tendon; the palmar wounds successfully healed with BTM however the dorsal wounds became infected and could not be salvaged despite IV antibiotics and our routine BTM salvage techniques. This patient ultimately required a central-slip reconstruction and local flap coverage to achieve healing. The exact cause of BTM failure in this is difficult to discern, as the thermal injury may have caused superficial tendon necrosis leading to poor vascularity and infection; additionally the location of wounds over mobile finger joints prevented to use of VAC therefore shearing of BTM may also have contributed to its failure.

Interestingly, in our case series 100 % of infected cases had exposed tendon in the wound base which raises some suspicion could this relate to the unrestricted mobility of underlying tendons creating dead space under BTM for bacteria to grow; a concept termed “pistoning” by Concannon et al.^17^ Such dead space may allow fluid collection, poor adherence of BTM to the bed and subsequent colonization and infection. This notion of a link between BTM loss and exposed tendon in the wound base is re-iterated by Grande et al. using univariate regression analysis to show a negative association (p < 0.001) between time to BTM integration and exposed tendon or muscle, as well as patient age.

Conversely, Tapking et al. reports no statistically significant correlation with injuries containing open joint or exposed tendon and BTM loss. Therefore further research is needed, focusing on BTM to reconstruct hand wounds and wounds with exposed tendon areas.

### Limitations

The studies included in the literature review were entirely observational, often retrospective and markedly heterogeneous. There was variability in the frequency of reporting all end-points, therefore the summary statistics are based on incomplete data and median values calculated were not weighted. Limitations of the consecutive case series were that documentation of dressings used over BTM in initial surgery was not always clear. The majority included Silver based dressings but many dressing types were not documented which would have been interesting to investigate if there was differential use between infected and non-infected groups. Pre-BTM swabs were not available for 23 % of patients but these may have been recorded or taken in referral hospitals prior to patient transfer. Additionally, this is an early case series, reflecting the first two years of experience with a new technology which creates a continuous learning curve of how best to utilize this technology. Our consecutive data collection is ongoing for all patients treated with BTM at our center and we hope that in future re-analysis, the rate of infection and BTM loss may decline with improvements in post-operative care and patient selection.

Despite these limitations, we believe the summary of the existing data, alongside our early cohort data, adds a valuable overview and context to the benefits and issues related to the use of BTM in the reconstruction of complex wounds.

## Conclusion

BTM is a useful reconstructive option especially in co-morbid patients with complex or infected wounds who may not be suitable candidates for flap reconstruction. It can also simplify a reconstructive problem, reducing a non-graftable bed to a graftable one. This study adds to the emerging body of evidence which suggests that, despite high rates of positive wound swabs for bacterial colonization, BTM infrequently becomes clinically infected. It therefore appears to be robust in the face of colonized wound surfaces and also can often be retained even following established localized infection.

Further research is required to explore the significance of positive wound swabs during the BTM integration phase, as the current evidence creates further questions around how clinicians should best manage pre-BTM positive wound swabs in clean wounds or whether they add any clinical value given that a key goal of BTM is to temporize wounds in a timely manner.

## Declaration of competing interest

The authors declare that they have no known competing financial interests or personal relationships that could have appeared to influence the work reported in this paper.

## Appendix Supplementary materials

Image, application 1
