# Supplementary material for: Biodegradable Temporizing Matrix (BTM) resilience to wound infection: A consecutive case series
Source: JPRAS Open. 2025 Aug 19;48:95–105. doi: 10.1016/j.jpra.2025.07.007 (PMC12688696; doi:10.1016/j.jpra.2025.07.007)
Supplement: Supplementary file 1 [file mmc1.docx]

| Author(s) and year | Journal | Title of paper | Study design | Patients in study analysis (n) | Wounds (n) | Average wound Size (TBSA vs cm^2^) | Average time from injury to BTM application (days) | Average time from debridement to BTM (days) | Average time from BTM to SSG (days) | Full BTM integration >95 % | Total BTM Loss (%) | Partial BTM Loss (%) | BTM wound swab positive (%) | BTM clinical infection (%) | Requiring SSG (%) | SSG wound swab positive (%) | SSG clinical infection (%) | Total SSG loss (%) | Partial SSG loss (%) |
| --- | --- | --- | --- | --- | --- | --- | --- | --- | --- | --- | --- | --- | --- | --- | --- | --- | --- | --- | --- |
| A Parker et al (2023) | European Journal of Plastic Surgery | The use of NovoSorb™ Biodegradable Temporising Matrix (BTM™) in the reconstruction of complex soft tissue defects — an oncological, aesthetic, and practical solution | Case series | 23 | 24 | <1 (%) | Not documented | Not documented | 43 | 95.8 | 4.2 | 0 | 58.8 | Not documented | 91.7 | 71.4 | Not documented | 8.9 | 4.2 |
| T Kidd et al (2023) | J Wound Care | The use of NovoSorb biodegradable temporising matrix in wound management: a literature review and case series. | Case series | 37 | 37 | 1 (%) | 8 | 6 | 53 | 70.3 | 24.3 | 5.4 | Not documented | 18.9 | 70.3 | Not documented | Not documented | Not documented | Not documented |
| F P Guerriero et al (2023) | Biomedicines | Overcoming Barriers to Wound Healing in a Neuropathic and Neuro-Ischaemic Diabetic Foot Cohort Using a Novel Bilayer Biodegradable Synthetic Matrix | Case series | 22 | 23 | Not documented | N/A | 0 | N/A | 65.3 | 34.7 | 0 | 52.1 | 13 | N/A | N/A | N/A | N/A | N/A |
| B Kuang (2022) | Scars Burns Heal | Use of Biodegradable Temporising Matrix (BTM) in the reconstruction of diabetic foot wounds: A pilot study | Case series | 14 | 14 | Not documented | N/A | Not documented | Not documented | 92.9 | 7.1 | 0 | Not documented | 14.3 | 28.6 | Not documented | Not documented | Not documented | Not documented |
| C Cheng (2021) | PRS Global Open | Reconstruction of Chronic Wounds Secondary to Injectable Drug Use with a Biodegradable Temporizing Matrix | Case series | 2 | 4 | 86 (cm^2^) | N/A | Not documented | 38.5 | 100 | 0 | 0 | 100 | 0 | 100 | Not documented | Not documented | Not documented | Not documented |
| Y Wu-Fienberg et al (2021) | PRS Global Open | An Alternative Dermal Template for Reconstruction of Complex Upper Extremity Wounds | Case series | 6 | 6 | 42 (cm^2^) | Not Documented | Not documented | Not documented | 100 | 0 | 0 | Not documented | 0 | 50 | Not documented | 0 | 0 | 0 |
| H Li (2021) | ANZ J Surg | Experience with NovoSorb® Biodegradable Temporising Matrix in reconstruction of complex wounds | Case series | 27 | 35 | Not documented | Not Documented | Not documented | 33 | 94.3 | 3.7 | 3.7 | Not documented | 0 | 91.4 | Not documented | Not documented | 3.1 | 12.5 |
| M Wagstaff (2019) | Burns Open | Biodegradable Temporising Matrix (BTM) for the reconstruction of defects following serial debridement for necrotising fasciitis: A case series | Case series | 7 | 7 | 8.6 (%) | 20.4 | Not documented | Not documented | 71.4 | 0 | 28.6 | Not documented | 0 | 100 | Not documented | 0 | 0 | 28.6 |
| M Wagstaff (2015) | E Plasty | Free Flap Donor Site Reconstruction: A Prospective Case Series Using an Optimized Polyurethane Biodegradable Temporizing Matrix | Case series | 10 | 10 | Not documented | 0 | Not documented | 36 | 100 | 0 | 0 | Not documented | 0 | 90 | Not documented | 0 | 0 | 0 |
|  |  |  |  |  |  |  |  |  |  |  |  |  |  |  |  |  |  |  |  |
| MEDIAN |  |  |  | 14 | 18.5 | <1% VS 91.5 cm^2^ | 14.2 | 3 | 38.5 | 94.3 | 3.7 | 0 | 58.8 | 0 | 90.7 | 71.4 | 0 | 0 | 4.2 |
| RANGE |  |  |  | 2 - 37 | 4 - 37 | <1%-24% VS 10 – 440 cm^2^ | 0 - 56 | 0-6 | 33 -53 | 65.3 - 100 | 0 - 34.7 | 0 - 28.6 | 52.1 - 100 | 0 - 18.9 | 28.6 - 100 | N/A | N/A | 0 -8.9 | 0 - 28.6 |
